# Ventilatory obstruction from kinked armoured tube

**DOI:** 10.4103/0019-5049.68380

**Published:** 2010

**Authors:** PS Balakrishna, Anil Shetty, Gayathri Bhat, US Raveendra

**Affiliations:** Department of Anaesthesiology and Critical Care, KSHEMA, Mangalore, India

Sir,

We share our experience of a kinked armoured endotracheal tube leading to difficulty in ventilation intra-operatively.

A 37 year-old male patient weighing 50 kg ASA I was posted for total thyroidectomy for papillary carcinoma of thyroid in euthyroid state. His vitals and sytemic examination were normal. Airway examination showed Modified Mallampati-II, Rules of 1,2,3 were normal. Investigations were within normal limits. After shifting the patient to operation theatre, baseline vitals were recorded - patient was premedicated with Inj Glycopyrrolate 0.2 mg and Inj Fentanyl 100 μg and preoxygenated. Induction was performed using Inj Propofol 100 mg; intubated with no. 8 cuffed armoured tube (ETO sterilized) with Inj Vecuronium 5 mg and secured after confirming equal bilateral air entry. Then the patient was connected to ventilator and ETCO_2_ monitor. Anaesthesia was maintained with IPPV with tidal volume 500 ml, Frequency 14/min with Nitrous Oxide: Oxygen 66:33 ratio, 1% Isoflurane and vecuronium top-ups. Patient was then placed in thyroid position, and surgery started.

**Figure 1:**
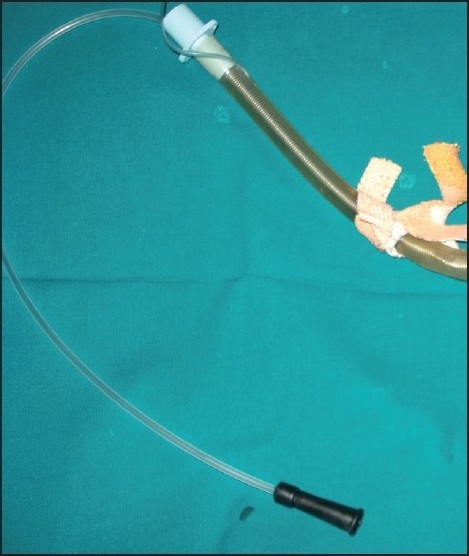
Unable to pass suction catheter

Approximately one and half hours after sugery, there was progressive increase in ETCO_2_ from 35 to 45 mmHg. Suspecting lighter planes, Isoflurane concentration was increased, vecuronium top up was repeated. Still ETCO_2_ increased and reached 50 mmHg. Patient’s vitals remained stable. Mechanical ventilation was changed over to manual ventilation. Ventilation was difficult and air entry was not made out. Acute bronchospasm was suspected. Nitrous Oxide was stopped, ventilation with 100% 02 resumed, Inj Deriphylline 1amp i.v, Inj Salbutamol 2.5 mg sc and Inj Hydrocortisone 100 mg i.v were given. Still the ventilation was difficult. Now we suspected the tube kink as a suction catheter attempted to pass through the ET tube got stuck in the mid-way [Figure 1]. Cuff was immediately deflated, Patient extubated and reintubated with a regular 7.5 cuffed PVC ET tube. Ventilation was possible with ease and surgery was allowed to continue.

**Figure 2:**
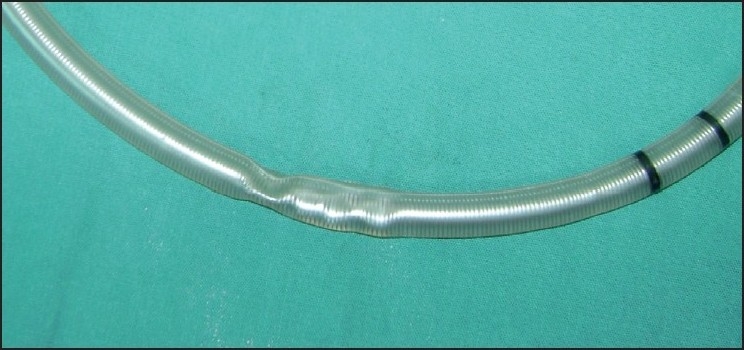
Tube with bite mark

**Figure 3:**
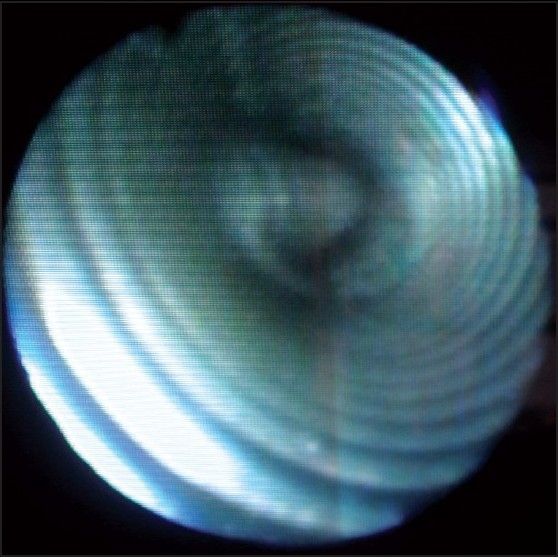
Fibre optic of the internal herniated cuff

A dent along the mid portion of the tube [Figure 2], fibre-optic view of the lumen, [Figure 3] confirmed presence of the kink. One probable reason for the kink could be a bite by the patient when he was under lighter planes as the kink corresponded to the patient’s lateral incisors.

Armoured tubes are better than conventional endotracheal tubes in Head and Neck,[1,2] Spine[3] and neuro-surgeries, because of the kink resistant nature, and flexibility even in its fixed position. But the disadvantages are cost factor, the need of stillete to insert the tube, difficulty in naso-tracheal insertion and inability to shorten the length, if needed.[3] There is a practice to re-use ETO sterlised tubes as in our set up. This makes the tube prone for kink and obstruction as in our case and other case reports.[4,5] Obstruction in flexo-metallic tubes could be due to secretions, blood,[6] aneurysmal cuff impinging the tip over tracheal lumen,[7] or kink at the shaft because of the bite as the patient becomes light[1–3,6] at the time of extubation. In our case, this kink occurred in the middle of surgery. Prompt diagnosis of this situation helped us avoid further complication like hypoxia, negative pressure pulmonary oedema and airway injury.
